# Our Co-Laborers

**Published:** 1878-01-20

**Authors:** 


### OUR CO LABORERS.

To those of our co-laborers for the past year, and all others who aided us by writing or otherwise, we are thankful. A number on the published list failed to do so, due, we suppose, to press of professional engagements, or other good reasons; while others not on the list have kindly interested themselves in our behalf.

Under these circumstances we have concluded to have no published list, but to ask all of our subscribers to consider themselves as our co-laborers, and to aid us in our efforts to benefit the profession and to develop the medical literature of our section.

---

Abingdon Academy of Medicine.—At the last annual meeting of this Association the following gentlemen were elected officers for the ensuing year:

Dr. Wm. L. Dunn, President.

Dr. J. S. Apperson, 1st Vice-President.

Dr. Geo. Ben. Johnston, 2d Vice-President.

Dr. Geo. E. Wiley, Recording Secretary.

Dr. W. F. Barr, Corresponding Secretary.

Dr. James Ogden, Treasurer.

Dr. William White, Essayist for the occasion, read an interesting essay on Dysmenorrhœa, which was requested to be furnished for publication.

Dr. E M. Campbell read a report of a case of a large fibroid tumor on the roof of the mouth, which he and Dr. G. B. Johnston, assisted by Dr. C. Aston, removed. It had been nearly thirty years growing, and the patient was not able to eat solids or talk much. The operation was successful.

Dr. W. F. Barr, reported a case of pelvic hæmatocele, occurring after parturition, successfully treated by himself.

The gentlemen were requested to furnish reports of their cases for publication.

Drs. J. F. Harrison and J. W. Mallet, of the University of Virginia; Dr. L. B. Edwards, editor of the Virginia Medical *Monthly;* Dr. J. L. Darby, of the University of New York; Surgeon General J. K. Barnes, of the United States army, Washington, D. C.; Dr. Greenville Dowell, of the Medical College of Texas, Galveston, Texas; Dr. R. L. Paynes, President of the Medical Society of North Carolina, Lexington, N. C.; and Prof. J. B. Reid, of Savannah, Ga., were elected Honorary Fellows of the Academy of Medicine.

---

Sample Copies.—Those who receive this number of The Record as a sample copy are requested to subscribe. Try our journal and see if it does not well deserve the name it has acquired of being the "favorite of the busy practitioner."

